# Evaluation of LDL goal achievement in statin consumption, south east of Iran

**DOI:** 10.1038/s41598-021-90228-0

**Published:** 2021-05-24

**Authors:** Malihe Aghasizadeh, Saeede Khosravi Bizhaem, Mahin Baniasadi, Mohammad Reza Khazdair, Toba Kazemi

**Affiliations:** 1 Student Research Committee, Department of Molecular Medicine, Faculty of Medicine, Birjand University of Medical Sciences, Birjand, Iran; 2 Cardiovascular Diseases Research Center, Birjand University of Medical Sciences, Birjand, Iran; 3 Student Research Committee, Birjand University of Medical Sciences, Birjand, Iran; 4 Razi Clinical Research Development Unit (RCRDU), Birjand University of Medical Sciences, Birjand, Iran

**Keywords:** Medical research, Drug development, Epidemiology

## Abstract

Lipid goal achievement and statin consumption were estimated at extreme/very-high/high/moderate and low cardiovascular risk categories. In the cross-sectional study, 585 patients treated with statin therapy referring to the heart clinic of Birjand were recruited. Patients were classified and examined LDL-C values and the proportion reaching targets according to the American Association of Clinical Endocrinologists guideline. Three patterns of statin use (high/moderate/low-intensity statin therapy) in all patients were examined and attainments of LDL-C goal in cardiovascular risk groups have been demonstrated. Over half the populations (57.6%) were in the very-high CVD risk group. The results showed that the proportion of patients meeting total LDL-C goal values according to the guidelines was 43.4%. The frequency of patient had achievement LDL goal lower in high-intensity pattern (N = 13, 2.3%), compared with moderate (N = 496, 86.1%) and low-intensity patterns (N = 67, 11.6%). In general, LDL-C goal achievement was greatest with moderate-intensity statin use. LDL-C reduction after statin consumption was estimated about one-third of the studied population. It seems likely that the achievement of a therapeutic target for serum lipids such as LDL-C improved is far more cost-effective and would be able to reach the target LDL as well changing the type and intensity of statins.

## Introduction

Cardiovascular disease (CVD) has been thought of as the first cause of death in non-communicable disorders with extends of urbanization and industrial lifestyle. CVD is affected by many factors such as age, hypertension, diabetes mellitus, dyslipidemia, insufficient activity, BMI, diet, and smoking^1^. Dyslipidemia is generally seen as a factor strongly related to CVD and elevated low-density lipoprotein cholesterol (LDL-C) has been recognized as the key lipid feature in these circumstances^2, 3^. Over the past few decades, statin derivatives have been used with safely and increasingly clinical advantage in keep away patient from the threat of CVD. These groups of inhibitors, by preventing the function of 3-hydroxy-3-methylglutaryl-coenzyme A (HMG-CoA) reductase, play an imperative role in reducing cholesterol and declining the risk of developing atherosclerosis plaques and heart attacks^4^.

Recent evidence suggests that a sharp decline in the levels of LDL-C that is obtained with statins not only increases lipid profiles improvement but also significantly prevented the incidence of cardiovascular events compared to conventional lipid-lowering treatments for high-risk patients^5^.This finding is supported by many scientists working on patients who were treated with a statin^6, 7^. Therefore, treatments with severe lipid reduction provide further clinical benefit and accelerate the improvement of atherosclerosis, which may lead to a decrease in cardiovascular events^8, 9^. However, the risk remains among statin-treated individuals and is known as "remaining risk"^10^. More recently, literature has emerged that offers contradictory findings of the effects of statin. Current guidelines focus on lowering LDL cholesterol as the main goal of statin therapy to reduce the risk of CVD^11^.

In 2013, statin guidelines in Canada and Europe supported the use of statin to achieve a steady-state of LDL with ≥ 50% reduction, whereas present US guidelines supporter the use of statin that decrease LDL Less and more than 50% in moderate and high intensity, respectively^6^. Attainment of therapeutic goals for serum lipids had recently been challenged by several studies demonstrating increasing LDL levels and the risk of cardiovascular disease in patients despite the use of statin even with high intensity^12–14^. One of the most significant current discussions in existing guidelines is different LDL target levels and lack of statin guidelines in Iran. This issue is a clinical problem that prompted studies to investigate LDL goals in patients treated with statins to improving patient follow-up and treatment policy.

Epidemiologic studies have shown that the risk classification of atherosclerotic cardiovascular disease to achieved LDL-C treatment goals agrees with the American Association of Clinical Endocrinologists (AACE) guideline in 2017. AACE presented clinical function guidelines for the administration of dyslipidemia and avoidance of CVD. The purpose of this article is to categorize the atherosclerotic cardiovascular diseases (ASCVD) risk level according to AACE guideline (2017) and estimate percentage of the patient treated with statin derivation for who have achieved the goal of treatment. Also, we examined clinically relevant patterns of statin use, based on statin type in a population with different levels of cardiovascular risk.

## Design study and method

In this cross-sectional study, a random sample of patients (576 number) treated with a statin (12 months) was recruited from the heart clinic of Birjand, who were 63.68 ± 9.96 years of age. After obtaining written informed consent from the participation, a questionnaire including personal data (height, weight, age, gender, marital status, occupational status, level of education, etc.) were completed and then people’s experiments and history of drug use were examined. In this descriptive-analytic design, the records of all patients treated with statin referring to the heart clinic of Birjand, the latest dose of statin, underlying disease, and lipid profile will be recorded by the researcher's checklist. Checklists also include a history of the disease (hypertension, diabetes, dyslipidemia, stroke, liver disease, hypothyroidism, hyperthyroidism, carotid stenosis, abdominal aneurysm), and biochemical tests (Chol, TG, HDL, LDL, FBS, urea, Cr, SGOT, SGPT, K, UA).

In this study, subgroups are divided according to cardiovascular and Framingham risk scoring was applied to determine 10 years risk factor can be defined as like extreme risk, very high risk, high risk, moderate risk, and low risk^15^. The first group was extreme risk who possesses progressive ASCVD or History of premature ASCVD, or CVD patient with DM, CKD 3/4, or HeFH. Also, individuals with type 2 diabetes (T2DM) should be considered as high, very high, or extreme risk for ASCVD. The main treatment goal of the current group was to achieve LDL-C < 55. Patients with progressive ASCVD including unstable angina or established clinical cardiovascular disease and individuals with DM, CKD 3/4, or HeFH and history of premature ASCVD (< 55 male, < 65 female) are proposed to be in the very high-risk category based AACE. In these patients, the treatment goal was defined as LDL-C < 70. High-risk patients were identified by ≥ 2 risk factors and 10-year risk 10–20% of patients with CKD 3/4 (with no other risk factors patients with DM), or HeFH history of premature ASCVD (< 55 male, < 65 female). LDL_C < 100 was identified as the goal for treatment in this subgroup. The Forth group (moderate risk) was included patients with ≤ 2 risk factors and 10-year risk < 10%. And the treatment goal was considered similar to high-risk patients. Finally, if there were any risk factors in the person, it was defined as low risk, and treatment goal was recommended to LDL-C < 130 in accordance with the guideline.

Statin derivation therapy could be low, moderate, or high intensity as shown in Table 1. High-intensity statins usually reduce LDL-C levels by 50% whereas moderate and low-intensity statins groups reduce LDL-C by 30–49% and less than 30%, respectively^16^. Percent LDL-C reductions with the derivation of statin medications (atorvastatin, simvastatin, rosuvastatin) used by the patient were assessed using the median reduction in LDL-C according to the VOYAGER database^17^.

**Table 1:** Definition of low-, moderate-, and high-intensity statin therapy and anticipated LDL-C reduction according to the 2017 American College of Cardiology/American Heart Association guideline.

	Low intensity statin	Moderate intensity statin	High intensity statin
LDL-C lowering	< 30%	30–49%	≥ 50%
Statin derivation	Simvastatin 10 mg Pravastatin 10–20 mg Lovastatin 20 mg Fluvastatin 20–40 mg Pitavastatin 1 mg	Atorvastatin 10–20 mg Rosuvastatin 5–10 mg Simvastatin 20–40 mg Pravastatin 40–80 mg Lovastatin 40 mg Fluvastatin XL 80 mg Fluvastatin 40 mg twice daily Pitavastatin 2–4 mg	Atorvastatin 40–80 mg Rosuvastatin 20–40 mg

Measurements of clinical parameters were performed as follows. To measure their blood pressure, they were asked to rest on a chair for at least 5 min, with their backs resting on the back of the chair with their arms approximately aligned with the heart. They also refrained from smoking tobacco or caffeine-containing substances at least 30 min before blood pressure was measured. A smoking period is defined the last month. Blood pressure was measured using an ALPK2 mercury barometer, cuff proportional to their arm circumference (such that the barometer covered two-thirds of the arm surface) from the right arm in sitting position, and recorded in a checklist. Weight was measured with minimum coverage and no shoes (coverage status for all individuals was equal using Balas model digital). Height was measured using a tape measure in a standing position without shoes while the shoulders were in normal condition. Waist circumference was measured in the thinnest area in which the individual was at the end of his natural exhaustion. The waist circumference was measured using a non-resilient meter, without imposing any pressure on the individual body with a precision of one millimeter. The body mass index is calculated using the formula (weight in kg divided by the square of height in square meters). According to WHO classification criteria, body mass index was less than 18.5 weight loss, 18.5 to 24.9 normal, 25.9 to 29.9 overweight, and greater than 30 obesity^18^.

An exclusion criterion of the study was low CVD risk groups; they were left out due to the small number of this group. Finally, since the dose of drugs is adjusted in three groups, so we also divided the groups in the table of guidelines that specify statin doses into three categories: Extreme and very high, high and moderate.

The manuscript was reviewed and approved by the Ethics Committee of Birjand University of Medical Sciences. Also, we confirmed that all methods were performed following the relevant guidelines and regulations.

### Statistical analysis

Data were entered into SPSS software (version 22) and analyzed using Chi-square (Fisher exact test), Mann–Whitney, and Kruskasl-Wallis tests as appropriate. Examine and descriptive statistics (frequency, mean, standard deviation, percentage Abundance) will be reported and the significance level of all tests will be considered at 5%. Since the distribution of variables based on the Kolmogorov–Smirnov test was not normal, so Mann–Whitney and Kruskal–Wallis tests were used for comparison in groups.

### Ethical approval and consent to participate

The written informed consent was signed by all the participants and the research was confirmed by the Ethics Committee of Birjand University of Medical Sciences. (Ir.bums.REC.1397.135).

### Consent for publication

Not applicable.

## Results

### Baseline characteristics of patients

576 statin-treated patients in Birjand were included in the study. All of the participants were aged 63.68 ± 9.96, of whom just over half the sample (N = 374, 64.9%) was female.529 (92.3%) of participation covering urban and 44 (7.7%) rural areas and only 15 (2.6%) of all patients had cigarette smoking. In terms of CVD risk factors, 451 (78.3%) had hypertension, 221 (38.4%) had diabetes, 232 (40.3%) had a BMI ≥ 30, 207 (36.1%) had triglycerides ≥ 150 mg/dL, 105 (18.2%) had total cholesterol ≥ 200 mg/dL,338 (58.7%) had HDL-C < 40 mg/dL in men and < 50 mg/dL in women (Low HDL).

**Figure 1:**
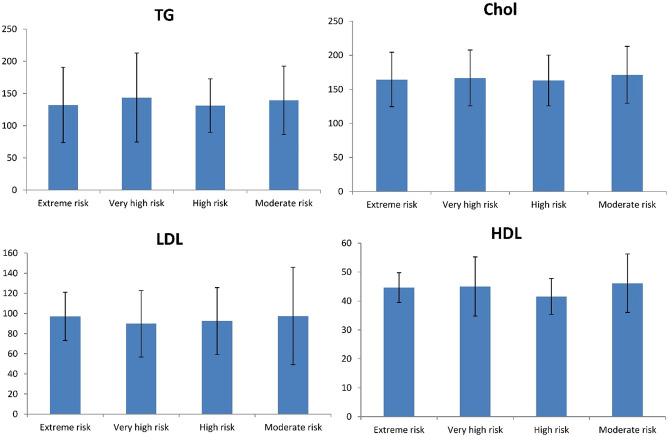
The value of lipid profile comparatively is drawn between the different ASCVD risk level category groups.

Precipitations were grouped according to the ASCVD risk level category based on AACE guidelines. The demographic and clinical characteristics of all patients are presented in Table 2. Demographic characteristics were variant little between cardiovascular risk groups. It is apparent from this table that patients in the very high-risk group had the highest number (n = 242) and the extreme groups were the lowest (n = 9). The data obtained from the lower risk group was not shown in the tables due to the small number of people in this group. As expected, individuals in the extreme risk group are older than the other groups. The proportion of the population with high ASCVD risk was significantly higher in age than in low-risk groups (p < 0.001). It is apparent from this table that there is a significant difference in other examined parameters (FBS, Cr, GFR, BMI, WC, smoking, and SBP) between the groups. So that, there is a difference in FBS between different groups; the lower risk group, the lower value of FBS. To better comprehend, the value of lipid profile (HDL, LDL, Total cholesterol, and TG) comparatively is drawn between the different ASCVD risk level category groups in Fig. 1. Further statistical tests revealed there were no significant differences between median total cholesterol and TG in the studied groups. Conversely, according to our result the significant difference in HDL-cholesterol between cardiovascular risk groups was found (p = 0.040).

**Table 2:** Demographic characteristics of 576 patients who treated with statin derivations.

Framingham risk variable	Risk category				
	Extreme risk (n = 9)	Very high risk (n = 337)	High risk (n = 55)	Moderate risk (n = 175)	*P* value
Age	73.56 ± 10.10	66.53 ± 9.89	64.55 ± 6.86	57.41 ± 7.70*^+#^	< 0.001
Female	9 (100%)	237 (70.3)	9 (16.4)	119 (68)	< 0.001
FBS	133.11 ± 52.14	127.57 ± 46.25	102.63 ± 12.25^+^	99.73 ± 11.71^+^	< 0.001
Cr	1 ± 0.2	1.06 ± 0.51	0.95 ± 0.2	0.89 ± 0.18^+^	< 0.001
GFR	55.02 ± 29.84	67.35 ± 29.75	85.4 ± 19.07*^+^	91.44 ± 21.27*^+^	< 0.001
BMI	27.91 ± 4.24	28.93 ± 5.49	28.86 ± 4.21	30.55 ± 4.39^+^	< 0.001
WC	97 ± 10.9	100.15 ± 10.96	102.69 ± 9.07	103.3 ± 9.07^+^	0.003
Smoker	1 (11.1)	8 (2.4)	5 (9.1)	1 (0.6)	0.004
SBP	143.89 ± 20.12	134.27 ± 16.10	133.36 ± 14.56	128.83 ± 14.89^+^	< 0.001
DBP	80 ± 10	78.87 ± 10.33	80.91 ± 10	79.48 ± 8.33	0.501

*Chol* total cholesterol, *TG* triglyceride, *FBS* fasting blood sugar, *BMI* body mass index, *WC *waist circumference, *SBP* systolic blood pressure, *DBP* diastolic blood pressure.

*Exterme risk versus other groups.

^+^Very high versus other groups.

^#^High versus other groups.

### Changes in LDL and LDL goal attainments

Table 3 shows attainments of goal LDL levels by risk groups. The total LDL-C (mean ± SD) value in the studied patient was 92.51 ± 38.36 mg/dL after statin consumption. In general, only 247 patients (42.9%) achieved the LDL-C target according to the AACE guideline. As shown in the Table 3 in terms of risk groups, the frequency of those who achieved target LDL was highest in the high-risk group (70.9%) and a greater proportion of patients who did not achieve target LDL were in the extreme risk group (100%). The percentage of studied patients who attained the target LDL is very high, and moderate risk was 95 (28.2), and 113 (64.6), respectively. As can be seen, from a very high-risk group to a moderate-risk group, LDL levels had been raised; also, the percentage of people who attained the LDL goals has increased.

**Table 3:** Proportion of patients attaining targets for low density lipoprotein (LDL) according to American Association of Clinical Endocrinologists (AACE) guideline.

			Risk category				Total
			Extreme risk (n = 9)	Very high risk (n = 337)	High risk (n = 55)	Moderate risk (n = 175)	
Goal LDL (ref)			< 55	< 70	< 100	< 100	
Total LDL			97.11 ± 23.97	89.81 ± 33.08	92.45 ± 33.26	97.5 ± 48.37	92.51 ± 38.36
Target LDL (ACC/AHA guideline)	No	N (%)	9 (100%)	242 (71.8)	16 (29.1)	62 (35.4)	329 (57.1)
	Yes	N (%)	0	95 (28.2)	39 (70.9)	113 (64.6)	247 (42.9)

*ACC/AHA* The American College of Cardiology/American Heart Association.

**Figure 2:**
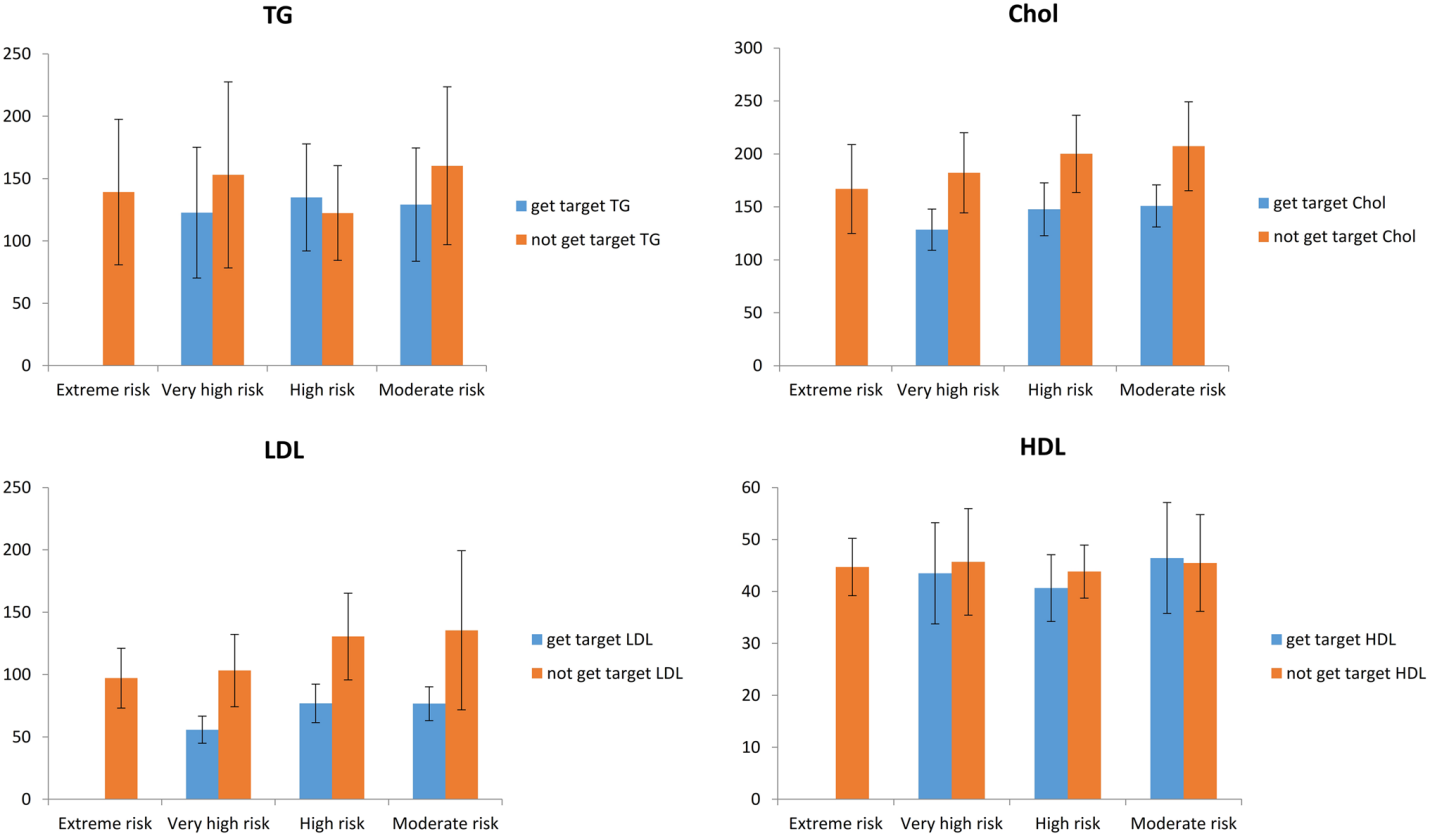
Comparison of lipid profile level between patients who get LDL goal and not achieved in different ASCVD risk level category groups.

We examined the demographic and clinical characteristics of these two groups (patients who get LDL goal and not achieved) separately in Tables 4 and 5. Comparison of lipid profile level (HDL, LDL, Total cholesterol, and TG) between patients who get LDL goal and not achieved in different ASCVD risk level category groups was shown in Fig. 2. We showed that level of cholesterol and LDL in a patient who get LDL goals and level of HDL in addition to the previous two lipids in patients who did not achieve were significantly different between ASCVD risk level category groups.

**Table 4:** Demographic characteristics of 329 patients who treated with statin derivations who did not get to target LDL.

Framingham risk variable	Risk category (not get to target LDL)				
	extreme risk (n = 9)	Very high risk (n = 242)	High risk (n = 16)	Moderate risk (n = 62)	*P *value
Age	73.88 ± 10.75	66.90 ± 9.39	63.44 ± 8.59	56.84 ± 7.85*^+^	< 0.001
Female	9 (100%)	186 (76.9%)	4 (25%)	52 (83.9%)	< 0.001
FBS	138.63 ± 52.86	128.45 ± 48.11	106.25 ± 11.7	100.32 ± 14.52^+^	< 0.001
Cr	0.95 ± 0.18	1.05 ± 0.55	0.92 ± 0.24	0.85 ± 0.16^+^	0.001
GFR	55.02 ± 29.84	66.22 ± 29. 37	86.91 ± 19.9^+^	90.97 ± 21.67*^+^	< 0.001
BMI	29.36 ± 4.31	28.66 ± 5.36	29 ± 4.34	30.72 ± 4.25^+^	0.014
WC	97.63 ± 11.48	99.32 ± 10.6	100.81 ± 7.08	102.74 ± 7.67^+^	0.047
Smoker	1 (11.1)	4 (1.7)	0	0	0.180
SBP	141.25 ± 19.78	135.13 ± 15.98	134.38 ± 16.92	129.83 ± 15	0.071
DBP	80 ± 10.69	79.19 ± 10.14	83.75 ± 12.04	80 ± 8.07	0.399

*Exterme risk versus other groups.

^+^Very high versus other groups.

^#^High versus other groups.

**Table 5:** Demographic characteristics of 247 patients who treated with statin derivations who achieved to target LDL.

Framingham risk variable	Risk category (get to target LDL)			
	Very high risk (n = 95)	High risk (n = 39)	Moderate risk (n = 113)	*P *value
Age	66.47 ± 10.35	65 ± 6.09	57.55 ± 7.77^+^^#^	< 0.001
Female	51 (53.7%)	5 (12.8%)	67 (59.3%)	< 0.001
FBS	126.51 ± 45.16	101.15 ± 12.3^+^	99.67 ± 10.13^+^	< 0.001
Cr	1.1 ± 0.46	0.96 ± 0.18	0.9 ± 0.2^+^	< 0.001
GFR	70.24 ± 30.68	84.78 ± 18.94^+^	91.7 ± 21.14^+^	< 0.001
BMI	29.05 ± 5.33	28.81 ± 4.22	30.33 ± 4.47	0.087
WC	101.36 ± 11.65	103.46 ± 9.75	103.21 ± 9.48	0.420
Smoker	4 (4.2)	5 (12.8)	1 (0.9)	0.006
SBP	133.01 ± 16.23	132.95 ± 13.7	128.3 ± 15.07	0.050
DBP	78.07 ± 10.71	79.74 ± 8.96	79.26 ± 8.56	0.597

*Exterme risk versus other groups.

^+^Very high versus other groups.

^#^High versus other groups.

### LDL goal and patterns of statin therapy

Traditionally, regarding statin derivation therapy guidelines, the distribution of individuals based on 4 cardiovascular risk groups was shown in Table 6. Somewhat frequency of patient had achieved LDL goal were lower in a high-intensity pattern 13 (2.3%), compared with moderate- and a low-intensity patterns; 496 (86.1%) and 67 (11.6%), respectively. In all groups, the most percentage of people who reached the target LDL use the moderate-intensity statin; this dosage of statin is recommended to achieve the LDL goal. According to statin derivation therapy guidelines, patients using the moderate-intensity of statin in extreme-, very high-, high-, and moderate-groups were 8 (88.9), 297 (88.1), 46 (83.6), and 145 (82.9), respectively. The percent of a patient with LDL goal who experiencing ≥ 50% reduction in LDL-C was very low (ranged from 1.5 to 3.6%). Table 7 had been demonstrated a comparison of individual distribution in CVD risk groups based on statin derivation therapy guideline between patients who get LDL goal and not achieved. According to this analysis, there was no association between statin derivation therapy and variant CVD risk group in either group who get LDL goal or who not achieved.

**Table 6:** Patterns of statin use and proportion of total patients in CVD risk groups.

Guidelines specify statin doses	Risk category				Total
	Extreme risk (n = 9)	Very high risk (n = 337)	High risk (n = 55)	Moderate risk (n = 175)	
High-intensity *↓ LDL-C by* ≥ *50%*	0	5 (1.5)	2 (3.6)	6 (3.4)	13 (2.3)
Moderate-intensity *↓LDL-C by 30–50%*	8 (88.9)	297 (88.1)	46 (83.6)	145 (82.9)	496 (86.1)
Low-intensity *↓ LDL-C by* < *30%*	1 (11.1)	35 (10.4)	7 (12.7)	24 (13.7)	67 (11.6)

Statin derivation therapy in three groups; low, moderate, or high intensity. High-intensity statins usually reduce LDL-C levels by 50% whereas moderate and low intensity statins groups reduce LDL-C by 30–49% and less than 30%, respectively.

**Table 7:** Comparison of statin patterns use and proportion of different CVD risk group in patients who get to target LDL and not achieved.

Guidelines specify statin doses	Risk category (Not get to target LDL)				Risk category (get to target LDL)			
	Extreme and very high	High risk	Moderate risk	*P* value	Extreme and very high	High risk	Moderate risk	*P* value
High-intensity *↓ LDL-C by* ≥ *50%*	5 (2)	1 (6.2)	4 (6.5)	0.063	0	1 (2.6)	2 (1.7)	0.642
Moderate-intensity *↓LDL-C by 30–50%*	223 (88.8)	12 (75)	49 (79)		82 (86.3)	34 (87.2)	96 (85)	
Low-intensity *↓ LDL-C by* < *30%*	23 (9.2)	3 (18.8)	9 (14.5)		13 (13.7)	4 (10.3)	15 (13.3)	

Statin derivation therapy in three groups; low, moderate, or high intensity. High-intensity statins usually reduce LDL-C levels by 50% whereas moderate and low intensity statins groups reduce LDL-C by 30–49% and less than 30%, respectively.

## Discussion

The present study categorized individual according to the atherosclerotic cardiovascular diseases (ASCVD) risk level and estimate percentage of patient achieved the LDL goal. Also, we examined clinically relevant patterns of statin use, based on statin type in a population with different levels of cardiovascular risk. In our study participants, 254 patients (43.4%) met their LDL-C goal. This level of LDL-C goal achievement is acceptable compared with parallel studies; Pearson (2000) identifies 38% of patients from United Stated who were treated with lipid-lowering for 3 months achieved LDL-C target level^19^. These results differ from Tomas’s 2006 estimate of 68% of patient’s attainment of LDL goals^20^. It seems possible that the period of statin therapy was different. The percentage of studied patients who attained the target LDL in Extreme risk, very high, high, and moderate risk was 0 (0), 95 (28.2), 39 (70.9), and 113 (64.6), respectively. With regards to assess of cardiovascular risk, about one-third of very high-risk patients and two-thirds of high and moderate-risk patients achieve their target goals. These results match those observed in earlier studies. Subsequent studies have reported 32.1% of the very high risk patients versus 51.9% and 55.7% of the high and moderate-risk patients achieved LDL-C goals^13^. These findings recommend that presently used statin derivation was insufficient to achieve LDL-C target in different risk groups and a gap exists among guideline recommendations and the use of treatments in this region. Our results showed that none of the patients with extreme cardiovascular risk attained the recommended LDL target; corresponding figures were 28.2% among high-risk patients and 70.9, 64.7, and 77.8% among high to low-risk patients. The study of LDL and cholesterol treatment goals was carried out by Svensson et al. They have been shown a reduction in median LDL and total cholesterol in statin users was 43 and 28%, respectively. The increased rate in percent of individuals achieved LDL goal among patients with very high-, high-, low to moderate-risk (54%, 82%, and 88%, respectively) had been observed^20, 21^.

For the first time, the results from the observational study on patients receiving LDL target in different risk groups show that the majority of these populations were in moderate-intensity statin therapy. The overall 86%, 87%, and 85%success percent for LDL-C goal attainment were in very high, high, and moderate risk patients. These data suggest that moderate-intensity statins therapy may be sufficient to achieve target goals in a percentage of all risk patients. A considerable amount of literature has been published on the high-risk cardiovascular populations. The retrospective cohort study showed that individuals with the low-intensity pattern were 3.9 of success person to earned LDL goal and the patient was treated by moderate and high-intensity pattern were about 72 and 30 percent of this participation^22^. On the other hand, the majority of people who achieved the LDL goal were treated with moderate-intensity statin medication, so it seems likely that by changing the type and dose of statins to the high-intensity statin therapy, these people would be able to reach the target LDL as well. Questions have been raised about the separate people who achieved LDL goals with specific diseases such as diabetes and CVD. Recent evidence suggests that 50.4% of patients with diabetes and 65.3% with vascular disease achieved an LDL-C level < 100 mg/dL^23^. In most recent studies, lipid-lowering drug toward LDL goal has been measured in different disease. Data for the study on chronic kidney disease (CKD)were demonstrated that only 30% of patients with CKD achieved the LDL-C goal regardless of the high intensity of statin treatment^24^. As noted by another paper achievement of the therapeutic target for serum lipids (LDL-C, HDL-C, and TG) improved is far more cost effective, in these investigations only two-third of patients achieve lipid goals. And about 60 patients did not get a goal for all targets (HDL-C, LDL-C, and TG). In our investigation there is not analysis on total cholesterol and HDL goals. It is therefore suggested that the target serum lipids profile be further investigated in future studies.

## Conclusion

In this investigation, the aim was to categorize the atherosclerotic cardiovascular diseases (ASCVD) risk level according to AACE guideline (2017) and estimate percentage of the patient treated with statin derivation for who have achieved the goal of treatment. These findings suggest that in general LDL-C goal attainment overall was 38%, and in detail about one-third of very high-risk patients and two-thirds of high and moderate-risk patients achieve their target goals. Taken together, the majority of people who achieved the LDL goal were treated with moderate-intensity statin medication. It would be expected that the “did not achieve LDL-C” group would have required treatment with statins of higher potency. Another possible area of future research would be to investigate other diseases and more lipid goals.

## Data Availability

Additional data are available from the corresponding authors for reasonable requesting.

## Abbreviations

ASCVD: Atherosclerotic cardiovascular disease
CVD: Cardiovascular disease
DM: Diabetes Mellitus
LDL-C: Low-density lipoprotein cholesterol
HMG-CoA: 3-Hydroxy-3-methylglutaryl-coenzyme A
AACE: American Association of Clinical Endocrinologists
T2DM: Type 2 diabetes mellitus
